# Mandibular ramus length and condylar changes in Juvenile Idiopathic Arthritis

**DOI:** 10.1186/1546-0096-9-S1-P172

**Published:** 2011-09-14

**Authors:** P Arvonen, J Naujokaityte, M Arvonen, ML Perkiömäki, P Vähäsalo, P Pirttiniemi

**Affiliations:** 1 University of Oulu, Finland; 2 University of Kaunas, Lithuania; 3 Pediatric Clinic, Kuopio University Hospital, Finland; 4 Pediatric Clinic, Oulu University Hospital. Finland

## Background

Universal recommendation of the evaluation of the temporomandibular joint (TMJ) involvements in juvenile idiopathic arthritis (JIA) is still not available.

## Aim

To assess the valuability of TMJ symtoms or findings as an indicator of mandibular involvement.

## Subjects and methods

62 (42 girls) out of 305 children with JIA aged 3-17 years and followed-up at the Paediatric Rheumatology OPD in Oulu University Hospital were involved in this cross-sectional study. Group 1 had new TMJ symptoms or findings and group 2 chronic TMJ symptoms or findings. Subjects were examined by a paediatric rheumatologist and by a dentist.The control group consisted of 68 healthy children (28 girls). We examined OPGs both from patients (mean age 10.4; range 3-17) and controls (9.3 years; range 6-14) and measured mandibular ramus lenght on OPG (the distance between point Condylion and point Gonion). We calculated the percential difference of the lenght of the contralateral ramus. Condylar changes were graded in five point scale (Billiau et al 2007).

## Results

Ramus lenght in age-matched scale was shorter in both JIA groups compared with controls. The mean persential asymmetry of the ramus was higher in both JIA groups. The corresponding asymmetry was ( 2.8% (range 0.1-9.9) in group 1, 4.3%, (0.1-12.7) in group 2,; and 1.2% (0- 5.8) in controls (p<0.025). The patients with early onset JIA had more severe TMJ changes and shorter length of the ramus than those with late onset JIA.

## Conclusion

In parallel with earlier studies, TMJ symptoms or findings were unsensitive indicators of mandibular involvements. Sensitive TMJ examinations should be accessible in all patients suffering from JIA including patients without TMJ symptoms.

